# Supplementary material for: Smooth Interpolating Curves with Local Control and Monotone Alternating Curvature
Source: Comput Graph Forum. 2022 Oct 6;41(5):25–38. doi: 10.1111/cgf.14600 (PMC9827861; doi:10.1111/cgf.14600)
Supplement: Supplementary file 1 — Supplement Material [file CGF-41-25-s001.zip › Local-Smooth-Interpolating-MonoCurvature/extern/clothoids/submodules/quarticRootsFlocke/docs/api-cpp/program_listing_file_PolynomialRoots-Jenkins-Traub.cc.html]

Program Listing for File PolynomialRoots-Jenkins-Traub.cc — Quartic Roots v1.0.0

### Navigation

- index
- toc
- Quartic Roots »
- Program Listing for File PolynomialRoots-Jenkins-Traub.cc

# Program Listing for File PolynomialRoots-Jenkins-Traub.cc¶

↰ Return to documentation for file (`PolynomialRoots-Jenkins-Traub.cc`)

```
//rpoly_ak1.cpp - Program for calculating the roots of a polynomial of real coefficients.
//Written in Visual C++ 2005 Express Edition
//14 July 2007
//
//The sub - routines listed below are translations of the FORTRAN routines included in RPOLY.FOR,
//posted off the NETLIB site as TOMS / 493:
//
//http: //www.netlib.org / toms / 493
//
//TOMS / 493 is based on the Jenkins - Traub algorithm.
//
//To distinguish the routines posted below from others, an _ak1 suffix has been appended to them.
//
//Following is a list of the major changes made in the course of translating the TOMS / 493 routines
//to the C++ versions posted below:
//1) All global variables have been eliminated.
//2) The "FAIL" parameter passed into RPOLY.FOR has been eliminated.
//3) RPOLY.FOR solves polynomials of degree up to 100, but      does    not explicitly state this limit.
//rpoly_ak1 explicitly states this limit; uses the macro name MAXDEGREE to specify this limit;

//and does a check to ensure that the user input variable Degree is not greater than MAXDEGREE
//(if it is, an error message is output and rpoly_ak1 terminates).If a user wishes to compute
//roots of polynomials of degree greater than MAXDEGREE, using a macro name like MAXDEGREE provides
//the simplest way of offering this capability.
//4) All "GO TO" statements have been eliminated.
//
//A small main program is included also, to provide an example of how to use rpoly_ak1.In this
//example, data is input from a file to eliminate the need for a user to type data in via
//the console.
//

#ifdef __GNUC__
#pragma GCC diagnostic ignored "-Wunused-function"
#endif
#ifdef __clang__
#pragma clang diagnostic ignored "-Wglobal-constructors"
#pragma clang diagnostic ignored "-Wvla-extension"
#pragma clang diagnostic ignored "-Wvla"
#pragma clang diagnostic ignored "-Wunused-function"
#pragma clang diagnostic ignored "-Wdocumentation-unknown-command"
#endif

#include "PolynomialRoots.hh"

#include <iostream>
#include <fstream>
#include <cctype>
#include <cmath>
#include <cfloat>
#include <limits>

#ifndef DOXYGEN_SHOULD_SKIP_THIS
using namespace std;
#endif

namespace PolynomialRoots {

  #ifndef DOXYGEN_SHOULD_SKIP_THIS

  using std::abs;
  using std::pow;
  using std::frexp;

  #ifndef M_PI
  #define M_PI 3.14159265358979323846264338328
  #endif

  //static valueType const maxValue   = std::numeric_limits<valueType>::max();
  //static valueType const minValue   = std::numeric_limits<valueType>::min();
  static valueType const epsilon    = std::numeric_limits<valueType>::epsilon();
  static valueType const epsilon10  = 10*epsilon;
  static valueType const epsilon100 = 100*epsilon;

  static valueType const RADFAC = M_PI / 180; // Degrees - to - radians conversion factor = pi / 180
  //static valueType const lb2    = log(2.0); // Dummy variable to avoid re - calculating this value in loop below
  static valueType const cosr   = cos(94.0 * RADFAC); //= -0.069756474
  static valueType const sinr   = sin(94.0 * RADFAC); //= 0.99756405

  //============================================================================
  // Divides p by the quadratic x^2+u*x+v
  // placing the quotient in q and the remainder in a, b
  static
  void
  QuadraticSyntheticDivision(
    indexType       NN,
    valueType       u,
    valueType       v,
    valueType const p[],
    valueType       q[],
    valueType     & a,
    valueType     & b
  ) {
    q[0] = b = p[0];
    q[1] = a = p[1] - (b*u);
    for ( indexType i = 2; i < NN; ++i )
      { q[i] = p[i]-(a*u+b*v); b = a; a = q[i]; }
  }

  //============================================================================
  // This routine calculates scalar quantities used to compute the next
  // K polynomial and new estimates of the quadratic coefficients.
  // calcSC - integer variable set here indicating how the calculations
  // are normalized to avoid overflow.
  static
  indexType
  calcSC(
    indexType       N,
    valueType       a,
    valueType       b,
    valueType     & a1,
    valueType     & a3,
    valueType     & a7,
    valueType     & c,
    valueType     & d,
    valueType     & e,
    valueType     & f,
    valueType     & g,
    valueType     & h,
    valueType const K[],
    valueType       u,
    valueType       v,
    valueType       qk[]
  ) {

    indexType dumFlag = 3; // TYPE = 3 indicates the quadratic is almost a factor of K

    // Synthetic division of K by the quadratic 1, u, v
    QuadraticSyntheticDivision( N, u, v, K, qk, c, d );

    if ( std::abs(c) <= epsilon100 * std::abs(K[N-1]) &&
         std::abs(d) <= epsilon100 * std::abs(K[N-2]) ) return dumFlag;

    h = v*b;
    if ( std::abs(d) >= std::abs(c) ) {
      dumFlag = 2; // TYPE = 2 indicates that all formulas are divided by d
      e  = a / d;
      f  = c / d;
      g  = u*b;
      a3 = e*(g+a) + h*(b/d);
      a1 = f*b - a;
      a7 = h + (f+u) * a;
    } else {
      dumFlag = 1; // TYPE = 1 indicates that all formulas are divided by c;
      e  = a/c;
      f  = d/c;
      g  = e*u;
      a3 = e*a + (g+h/c)*b;
      a1 = b - (a*(d/c));
      a7 = g*d + h*f + a;
    }
    return dumFlag;
  }

  //============================================================================
  // Computes the next K polynomials using the scalars computed in calcSC_ak1
  static
  void
  nextK(
    indexType       N,
    indexType       tFlag,
    valueType       a,
    valueType       b,
    valueType       a1,
    valueType     & a3,
    valueType     & a7,
    valueType       K[],
    valueType const qk[],
    valueType const qp[]
  ) {
    if ( tFlag == 3 ) {
      // Use unscaled form of the recurrence
      K[1] = K[0] = 0;
      for ( indexType i = 2; i < N; ++i ) K[i] = qk[i-2];
    } else {
      valueType temp = tFlag == 1 ? b : a;
      if ( std::abs(a1) > epsilon10 * std::abs(temp) ) {
        // Use scaled form of the recurrence
        a7 /= a1;
        a3 /= a1;
        K[0] = qp[0];
        K[1] = qp[1]-(a7*qp[0]);
        for ( indexType i = 2; i < N; ++i )
          K[i] = qp[i] - (a7*qp[i-1]) + a3*qk[i-2];
      } else {
        // If a1 is nearly zero, then use a special form of the recurrence
        K[0] = 0;
        K[1] = -a7 * qp[0];
        for ( indexType i = 2; i < N; ++i )
          K[i] = a3*qk[i-2] - a7*qp[i-1];
      }
    }
  }

  //============================================================================
  // Compute new estimates of the quadratic coefficients
  // using the scalars computed in calcSC
  static
  void
  newest(
    indexType       tFlag,
    valueType     & uu,
    valueType     & vv,
    valueType       a,
    valueType       a1,
    valueType       a3,
    valueType       a7,
    valueType       b,
    valueType       c,
    valueType       d,
    valueType       f,
    valueType       g,
    valueType       h,
    valueType       u,
    valueType       v,
    valueType const K[],
    indexType       N,
    valueType const p[]
  ) {

    vv = uu = 0; // The quadratic is zeroed
    if ( tFlag != 3 ) {
      valueType  a4, a5;
      if (tFlag != 2) {
        a4 = a + u * b + h * f;
        a5 = c + (u + v * f) * d;
      } else {
        a4 = (a + g) * f + h;
        a5 = (f + u) * c + v * d;
      }
      // Evaluate new quadratic coefficients
      valueType b1 = -K[N - 1] / p[N];
      valueType b2 = -(K[N - 2] + b1 * p[N - 1]) / p[N];
      valueType c1 = v * b2 * a1;
      valueType c2 = b1 * a7;
      valueType c3 = b1 * b1 * a3;
      valueType c4 = -(c2 + c3) + c1;
      valueType temp = -c4 + a5 + b1 * a4;
      if ( temp != 0.0) {
        uu = -((u * (c3 + c2) + v * (b1 * a1 + b2 * a7)) / temp) + u;
        vv = v * (1.0 + c4 / temp);
      }
    }
  }

  //============================================================================
  // Variable - shift H - polynomial iteration for a real zero
  // sss - starting iterate
  // NZ - number of zeros found
  // iFlag - flag to indicate a pair of zeros near real axis
  static
  void
  RealIT(
    indexType     & iFlag,
    indexType     & NZ,
    valueType     & sss,
    indexType       N,
    valueType const p[],
    indexType       NN,
    valueType       qp[],
    valueType     & szr,
    valueType     & szi,
    valueType       K[],
    valueType       qk[]
  ) {
    iFlag = NZ = 0;
    valueType t=0, omp=0, s = sss;
    for (indexType j=0;;) {
      valueType pv = p[0]; // Evaluate p at s
      qp[0] = pv;
      for ( indexType i = 1; i < NN; ++i )
        qp[i] = pv = pv * s + p[i];
      valueType mp = std::abs(pv);
      // Compute a rigorous bound on the error in evaluating p
      valueType ms = std::abs(s);
      valueType ee = 0.5 * std::abs(qp[0]);
      for ( indexType i = 1; i < NN; ++i ) ee = ee * ms + std::abs(qp[i]);
      // Iteration has converged sufficiently if the polynomial
      // value is less than 20 times this bound
      if ( mp <= 20.0 * epsilon * (2*ee - mp) )
        { NZ = 1; szr = s; szi = 0; break; }

      // Stop iteration after 10 steps
      if ( ++j > 10 ) break;
      if ( j >= 2 ) {
        if ( (abs(t) <= 0.001 * std::abs(s-t)) && (mp > omp) ) {
          // A cluster of zeros near the real axis has been encountered;
          // Return with iFlag set to initiate a quadratic iteration
          iFlag = 1;
          sss   = s;
          break;
        }
      }
      // Return if the polynomial value has increased significantly
      omp = mp;
      // Compute t, the next polynomial and the new iterate
      valueType kv = qk[0] = K[0];
      for ( indexType i = 1; i < N; ++i )
        qk[i] = kv = kv * s + K[i];

      if ( std::abs(kv) > std::abs(K[N-1]) * epsilon10 ) {
        // Use the scaled form of the recurrence if the value of K at s is non - zero
        valueType tt = -(pv / kv);
        K[0] = qp[0];
        for ( indexType i = 1; i < N; ++i ) K[i] = tt * qk[i-1] + qp[i];
      } else { // Use unscaled form
        K[0] = 0.0;
        for ( indexType i = 1; i < N; ++i ) K[i] = qk[i-1];
      }
      kv = K[0];
      for ( indexType i = 1; i < N; ++i ) kv = kv * s + K[i];
      t = std::abs(kv) > std::abs(K[N-1])*epsilon10 ? -(pv/kv) : 0;
      s += t;
    }
  }

  //============================================================================
  // Variable - shift K - polynomial iteration for a quadratic
  // factor converges only if the zeros are equimodular or nearly so.
  static
  void
  QuadIT(
    indexType       N,
    indexType     & NZ,
    valueType       uu,
    valueType       vv,
    valueType     & szr,
    valueType     & szi,
    valueType     & lzr,
    valueType     & lzi,
    valueType       qp[],
    indexType       NN,
    valueType     & a,
    valueType     & b,
    valueType const p[],
    valueType       qk[],
    valueType     & a1,
    valueType     & a3,
    valueType     & a7,
    valueType     & c,
    valueType     & d,
    valueType     & e,
    valueType     & f,
    valueType     & g,
    valueType     & h,
    valueType       K[]
  ) {

    NZ = 0; // Number of zeros found
    indexType   j = 0, tFlag;
    valueType u = uu; // uu and vv are coefficients of the starting quadratic
    valueType v = vv;
    valueType relstp = 0; // initialize remove warning
    valueType omp    = 0; // initialize remove warning
    valueType ui, vi;
    bool triedFlag = false;
    do {
      Quadratic solve( 1.0, u, v );
      solve.getRoot0(szr,szi);
      solve.getRoot1(lzr,lzi);

      // Return if roots of the quadratic are real and not close
      // to multiple or nearly equal and of opposite sign.
      if ( std::abs(abs(szr) - std::abs(lzr)) > 0.01 * std::abs(lzr) ) break;
      // Evaluate polynomial by quadratic synthetic division
      QuadraticSyntheticDivision( NN, u, v, p, qp, a, b );
      valueType mp = std::abs(a-(szr*b)) + std::abs(szi*b);
      // Compute a rigorous bound on the rounding error in evaluating p
      valueType zm = std::sqrt(abs(v));
      valueType ee = 2 * std::abs(qp[0]);
      valueType t  = -szr*b;
      for ( indexType i = 1; i < N; ++i ) ee = ee * zm + std::abs(qp[i]);
      ee = ee * zm + std::abs(a+t);
      ee = (9*ee+2*abs(t)-7*(abs(a+t)+zm*abs(b)))*epsilon;
      // Iteration has converged sufficiently if the polynomial
      // value is less than 20 times this bound
      if ( mp <= 20*ee ) { NZ = 2; break; }
      // Stop iteration after 20 steps
      if ( ++j > 20 ) break;
      if ( j >= 2 ) {
        if ( (relstp <= 0.01) && (mp >= omp) && !triedFlag ) {
          // A cluster appears to be stalling the convergence.
          // Five fixed shift steps are taken with a u, v close to the cluster.
          relstp = (relstp < epsilon) ? std::sqrt(epsilon) : std::sqrt(relstp);
          u -= u * relstp;
          v += v * relstp;
          QuadraticSyntheticDivision(NN, u, v, p, qp, a, b);
          for ( indexType i = 0; i < 5; ++i ) {
            tFlag = calcSC(N, a, b, a1, a3, a7, c, d, e, f, g, h, K, u, v, qk);
            nextK(N, tFlag, a, b, a1, a3, a7, K, qk, qp);
          }
          triedFlag = true;
          j = 0;
        }
      }
      omp = mp;
      // Calculate next K polynomial and new u and v
      tFlag = calcSC(N, a, b, a1, a3, a7, c, d, e, f, g, h, K, u, v, qk);
      nextK(N, tFlag, a, b, a1, a3, a7, K, qk, qp);
      tFlag = calcSC(N, a, b, a1, a3, a7, c, d, e, f, g, h, K, u, v, qk);
      newest(tFlag, ui, vi, a, a1, a3, a7, b, c, d, f, g, h, u, v, K, N, p);

      // If vi is zero, the iteration is not converging
      if ( !isZero(vi) ) {
        relstp = std::abs((vi-v)/vi);
        u = ui;
        v = vi;
      }
    } while( !isZero(vi) );
  }

  //============================================================================
  /*
  // Computes up to L2 fixed shift K - polynomials, testing for
  // convergence in the linear or quadratic case.Initiates one of
  // the variable shift iterations and returns with the number of zeros found.
  //
  // L2 limit of fixed shift steps
  // NZ number of zeros found
  */
  static
  indexType
  FixedShift(
    indexType   L2,
    valueType   sr,
    valueType   v,
    valueType   K[],
    indexType   N,
    valueType   p[],
    indexType   NN,
    valueType   qp[],
    valueType   u,
    valueType & lzi,
    valueType & lzr,
    valueType & szi,
    valueType & szr
  ) {

    #ifdef _MSC_VER
    valueType * qk  = (valueType*)alloca( 2*(N+1)*sizeof(valueType) );
      valueType * svk = qk+N+1;
    #else
    valueType   qk[N+1], svk[N+1];
      #endif

    indexType iFlag = 1;
    indexType NZ    = 0;
    valueType betav = 0.25;
    valueType betas = 0.25;
    valueType oss   = sr;
    valueType ovv   = v;

    // Evaluate polynomial by synthetic division
    valueType a, b;
    QuadraticSyntheticDivision(NN, u, v, p, qp, a, b);
    valueType a1, a3, a7, c, d, e, f, g, h;
    indexType tFlag =   calcSC(N, a, b, a1, a3, a7, c, d, e, f, g, h, K, u, v, qk);

    valueType otv = 0; // initialize remove warning
    valueType ots = 0; // initialize remove warning
    for ( indexType j = 0; j < L2; ++j ) {
      indexType fflag = 1;
      // Calculate next K polynomial and estimate v
      nextK( N, tFlag, a, b, a1, a3, a7, K, qk, qp );
      tFlag = calcSC(N, a, b, a1, a3, a7, c, d, e, f, g, h, K, u, v, qk);
      valueType ui, vi;
      newest(tFlag, ui, vi, a, a1, a3, a7, b, c, d, f, g, h, u, v, K, N, p);
      valueType vv = vi;
      // Estimate s
      valueType ss = (K[N-1] != 0.0) ? -(p[N]/K[N-1]) : 0.0;
      valueType ts = 1.0;
      valueType tv = 1.0;
      if ( (j != 0) && (tFlag != 3) ) {
        // Compute relative measures of convergence of s and v sequences
        tv = (vv != 0.0) ? std::abs((vv - ovv) / vv) : tv;
        ts = (ss != 0.0) ? std::abs((ss - oss) / ss) : ts;
        // If decreasing, multiply the two most recent convergence measures
        valueType tvv = (tv < otv) ? tv * otv : 1;
        valueType tss = (ts < ots) ? ts * ots : 1;
        // Compare with convergence criteria
        bool vpass = tvv < betav;
        bool spass = tss < betas;
        if ( spass || vpass ) {
          // At least one sequence has passed the convergence test.
          // Store variables before iterating
          for ( indexType i = 0; i < N; ++i ) svk[i] = K[i];
          valueType s = ss;
          // Choose iteration according to the fastest converging sequence
          indexType stry = 0;
          indexType vtry = 0;
          for (;;) {
            if ( (fflag && ((fflag = 0) == 0)) &&
                 ((spass) && (!vpass || (tss < tvv))) ) {
                      ;
                        //Do nothing.Provides a quick "short circuit".
            } else {
              QuadIT(N, NZ, ui, vi, szr, szi, lzr, lzi, qp, NN, a, b, p, qk, a1, a3, a7, c, d, e, f, g, h, K);
              if ( NZ > 0) return NZ;
              // Quadratic iteration has failed.Flag that it has been
              // tried and decrease the convergence criterion
              iFlag = vtry = 1;
              betav *= 0.25;
              // Try linear iteration if it has not been tried and the s sequence is converging
              if ( stry || !spass ) iFlag = 0;
              else                  std::copy( svk, svk + N, K );
            }
            if ( iFlag != 0 ) {
              RealIT(iFlag, NZ, s, N, p, NN, qp, szr, szi, K, qk);
              if ( NZ > 0 ) return NZ;
              // Linear iteration has failed.Flag that it has been
              // tried and decrease the convergence criterion
              stry = 1;
              betas *= 0.25;
              if (iFlag != 0) {
                // If linear iteration signals an almost double real zero,                                attempt   quadratic iteration
                ui = -(s + s);
                vi = s * s;
                continue;
              }
            }
            // Restore variables
            std::copy( svk, svk+N, K );

            // Try quadratic iteration if it has not been tried
            // and the v sequence is converging
            if (!vpass || vtry) break; // Break out of infinite for loop
          }
          // Re - compute qp and scalar values to continue the second stage
          QuadraticSyntheticDivision(NN, u, v, p, qp, a, b);
          tFlag = calcSC(N, a, b, a1, a3, a7, c, d, e, f, g, h, K, u, v, qk);
        }
      }
      ovv = vv;
      oss = ss;
      otv = tv;
      ots = ts;
    }
    return NZ;
  }

  //============================================================================

  static
  inline
  valueType
  evalPoly( valueType x, valueType const p[], indexType Degree ) {
    valueType ff = p[0];
    for ( indexType i = 1; i <= Degree; ++i ) ff = ff * x + p[i];
    return ff;
  }

  //============================================================================

  static
  inline
  void
  evalPoly(
    valueType       x,
    valueType const p[],
    indexType       Degree,
    valueType     & f,
    valueType     & df
  ) {
    df = f = p[0];
    for ( indexType i = 1; i < Degree; ++i ) {
      f  = x * f + p[i];
      df = x * df + f;
    }
    f = x * f + p[Degree];
  }

  //============================================================================

  /*
  // Scale if there are large or very small coefficients
  // Computes a scale factor to multiply the coefficients of the polynomial.
  // The scaling is done to avoid overflow and to avoid undetected underflow
  // interfering with the convergence criterion.
  // The factor is a power of the base.
  */
  static
  inline
  void
  scalePoly( valueType p[], indexType N ) {
    /*
    .. double ldexp(double x, int n)
    .. The ldexp() functions multiply x by 2 to the power n.
    ..
    .. double frexp(double value, int *exp);
    .. The frexp() functions break the floating-point number value into
    .. a normalized fraction and an integral power of 2.
    .. They store the integer in the int object pointed to by exp.
    .. The functions return a number x such that x has a magnitude in
    .. the interval [1/2, 1) or 0, and value = x*(2**exp).
    */
    int max_exponent = std::numeric_limits<int>::min();
    for ( int i = 0; i <= N; ++i ) {
      if ( !isZero(p[i]) ) {
        int exponent;
        frexp( p[i], &exponent );
        if ( exponent > max_exponent ) max_exponent = exponent;
      }
    }
    int l = -max_exponent;
    for ( indexType i = 0; i <= N; ++i ) p[i] = ldexp(p[i],l);
  }

  //============================================================================

  // Compute lower bound on moduli of zeros
  static
  inline
  valueType
  lowerBoundZeroPoly( valueType p[], indexType N ) {

    #ifdef _MSC_VER
    valueType * pt  = (valueType*)alloca( (N+1)*sizeof(valueType) );
    #else
    valueType pt[N+1];
      #endif

    for ( indexType i = 0; i < N; ++i ) pt[i] = std::abs(p[i]);
    pt[N] = -abs(p[N]);

    // Compute upper estimate of bound
    valueType x = exp((log(-pt[N]) - log(pt[0]))/N);
    if ( !isZero(pt[N-1]) ) { // If Newton step at the origin is better, use it
      valueType xm = -pt[N]/pt[N-1];
      if ( xm < x ) x = xm;
    }
    // Chop the interval(0, x) until f <= 0
    valueType xm = x;
    while ( evalPoly( xm, pt, N ) > 0 ) { x = xm; xm = 0.1 * x; }

    // Do Newton iteration until x converges to two decimal places
    valueType dx;
    do {
      valueType f, df;
      evalPoly( x, pt, N, f, df );
      dx = f / df;
      x -= dx;
    } while ( std::abs(dx) > std::abs(x)*0.005 );
    return x;
  }

  //============================================================================

  static
  inline
  void
  roots3(
    valueType const p[4],
    indexType       Degree,
    valueType       zeror[],
    valueType       zeroi[]
  ) {
    if ( Degree == 1 ) {
      zeror[0] = -(p[1]/p[0]);
      zeroi[0] = 0;
    } else if ( Degree == 2 ) {
      Quadratic solve( p[0], p[1], p[2] );
      switch ( solve.numRoots() ) {
        case 2: solve.getRoot1( zeror[1], zeroi[1] );
        case 1: solve.getRoot0( zeror[0], zeroi[0] );
      }
    } else if ( Degree == 3 ) {
      Cubic solve( p[0], p[1], p[2], p[3] );
      switch ( solve.numRoots() ) {
        case 3: solve.getRoot2( zeror[2], zeroi[2] );
        case 2: solve.getRoot1( zeror[1], zeroi[1] );
        case 1: solve.getRoot0( zeror[0], zeroi[0] );
      }
    }
  }

  #endif

  //============================================================================
  int
  roots(
    valueType const * op,
    indexType         Degree,
    valueType       * zeror,
    valueType       * zeroi
  ) {

    if ( Degree < 1 ) return -1;

    // Do a quick check to see if leading coefficient is 0
    // The leading coefficient is zero. No further action taken. Program terminated
    if ( isZero(op[0]) ) return -2;

    #ifdef _MSC_VER
    valueType * ptr = (valueType*)alloca( 4*(Degree+1)*sizeof(valueType) );
    valueType * K    = ptr; ptr += Degree+1;
    valueType * p    = ptr; ptr += Degree+1;
    valueType * qp   = ptr; ptr += Degree+1;
    valueType * temp = ptr; ptr += Degree+1;
    #else
    valueType K[Degree+1];
    valueType p[Degree+1];
    valueType qp[Degree+1];
    valueType temp[Degree+1];
    #endif

    int N = Degree;
    valueType xx = std::sqrt(0.5); //= 0.70710678
    valueType yy = -xx;

    // Remove zeros at the origin, if any
    for ( indexType j = 0; isZero(op[N]); ++j, --N ) zeror[j] = zeroi[j] = 0.0;
    std::copy( op, op+N+1, p ); // Make a copy of the coefficients

    while ( N > 0 ) {
      // Main loop
      // Start the algorithm for one zero
      if ( N < 4 ) { roots3( p, N, zeror+Degree-N, zeroi+Degree-N ); break; }

      // Find the largest and smallest moduli of the coefficients
      scalePoly( p, N );

      // Compute lower bound on moduli of zeros
      valueType bnd = lowerBoundZeroPoly( p, N );

      // Compute the derivative as the initial K polynomial and
      // do 5 steps with no shift
      for ( indexType i = 1; i < N; ++i ) K[i] = ((N-i) * p[i]) / N;
      K[0] = p[0];
      indexType NM1 = N-1;
      valueType aa = p[N];
      valueType bb = p[NM1];
      bool zerok = isZero(K[NM1]);
      for ( indexType iter = 0; iter < 5; ++iter ) {
        if ( zerok ) { // Use unscaled form of recurrence
          for ( indexType i = 0; i < NM1; ++i ) K[NM1-i] = K[NM1-i-1];
          K[0] = 0;
          zerok = isZero(K[NM1]);
        } else { // Used scaled form of recurrence if value of K at 0 is nonzero
          valueType t = -aa / K[NM1];
          for ( indexType i = 0; i < NM1; ++i ) {
            indexType j = NM1-i;
            K[j] = t * K[j-1] + p[j];
          }
          K[0] = p[0];
          zerok = std::abs(K[NM1]) <= std::abs(bb) * epsilon10;
        }
      }

      // Save K for restarts with new shifts
      std::copy( K, K+N, temp );

      // Loop to select the quadratic corresponding to each new shift
      bool ok = false;
      for ( indexType iter = 0; iter < 20 && !ok; ++iter ) {
        // Quadratic corresponds to a double shift to a non-real point and its
        // complex conjugate. The point has modulus BND and amplitude rotated
        // by 94 degrees from the previous shift.
        valueType tmp = -(sinr * yy) + cosr * xx;
        yy = sinr * xx + cosr * yy;
        xx = tmp;
        valueType sr = bnd * xx;
        valueType u = -2*sr;
        // Second stage calculation, fixed quadratic
        valueType lzi, lzr, szi, szr;
        indexType NZ = FixedShift( 20*(iter+1), sr, bnd, K, N, p, N+1, qp, u, lzi, lzr, szi, szr);
        ok = NZ != 0;
        if ( ok ) {
          //The second stage jumps directly to one of the third stage iterations and
          //returns here if successful.Deflate the polynomial, store the zero or
          //zeros, and return to the main algorithm.
          indexType j = Degree - N;
          zeror[j] = szr;
          zeroi[j] = szi;
          N -= NZ;
          for ( indexType i = 0; i <= N; i++) p[i] = qp[i];
          if ( NZ != 1 ) {
            zeror[j+1] = lzr;
            zeroi[j+1] = lzi;
          }
        } else {
          // If the iteration is unsuccessful, another quadratic is chosen after restoring K
          std::copy( temp, temp+N, K );
        }
      }
      // Return with failure if no convergence with 20 shifts
      if ( !ok ) return -2;
    }
    return 0;
  }
}
```

### Quick search

### Table of Contents

- Quartic Roots Solver
- C++ API

«
hide menu

menu
sidebar
»

### Navigation

- index
- toc
- Quartic Roots »
- Program Listing for File PolynomialRoots-Jenkins-Traub.cc

© Copyright 2021, Enrico Bertolazzi.
Created using Sphinx 4.2.0.
